# Development of a novel, short, self-completed questionnaire on empowerment for patients with type 2 diabetes mellitus and an analysis of factors affecting patient empowerment

**DOI:** 10.1186/1751-0759-8-19

**Published:** 2014-08-25

**Authors:** Yoriko Hara, Sanae Iwashita, Akira Okada, Yuji Tajiri, Hitomi Nakayama, Tomoko Kato, Motoyuki Nakao, Koji Tsuboi, Raoul Breugelmans, Yoko Ishihara

**Affiliations:** 1 School of Nursing, Kurume University, Higashikushiharamachi 777-1, Kurume, Fukuoka 830-0003, Japan; 2 Chidoribashibyouin Sue Clinic, Fukuoka, Japan; 3 Okada Clinic, Fukuoka, Japan; 4 Department of Endocrinology and Metabolism, School of Medicine, Kurume University, Fukuoka, Japan; 5 Department of Public Health, School of Medicine, Kurume University, Fukuoka, Japan; 6 Department of Psychosomatic Medicine, School of Medicine, Toho University, Tokyo, Japan; 7 Department of Medical Education, Tokyo Medical University, Tokyo, Japan

**Keywords:** Type 2 diabetes mellitus, Empowerment questionnaire, Appraisal of diabetes scale, Diabetes family behavior checklist, Self-managed behavior

## Abstract

**Background:**

Patient empowerment has recently been proposed as an important concept in self-management for effective glycemic control. A concise self-completed questionnaire for patients with type 2 diabetes mellitus was created to comprehensively evaluate their empowerment on the basis of self-managed dietary/exercise behaviors, psychological impact, and family support. The reliability and validity of this short questionnaire were tested and factors relating to patient empowerment were analyzed.

**Methods:**

The self-completed empowerment questionnaire was based on questionnaires for self-managed dietary and exercise behaviors, the Appraisal of Diabetes Scale, and the Diabetes Family Behavior Checklist. The questionnaire was trialed on 338 male and female patients with type 2 diabetes mellitus who lived with family. The validity and reliability of the questionnaire were investigated and stepwise multiple linear regression analysis was used to identify factors that affect patient empowerment.

**Results:**

The self-completed patient empowerment questionnaire included 13 questions on background data (e.g., age, gender, and HbA1c) and 18 questions within five scales to assess self-managed dietary behaviors, self-managed exercise behaviors, and psychological impact of diabetes, as well as positive and negative feedback in patient-family communication. The questionnaire showed sufficient internal consistency, construct validity, reproducibility, factorial construct validity, and concurrent validity. The results were generally satisfactory, and the questionnaire reflected the particular characteristics of treatment methods. Multiple linear regression analysis showed that patient empowerment was strongly affected by the number of disease-related symptoms, age, and gender.

**Conclusions:**

The results suggest that the concise self-completed empowerment questionnaire developed here is useful for measuring the empowerment of individual patients and evaluating the impact of symptoms and therapies on empowerment.

## Background

Current estimates suggest that as of 2013 there were 380 million patients with diabetes mellitus worldwide and that healthcare spending on this disease and its complications had risen to $548 billion [1]. Owing to changing dietary habits and demographic aging, over 9.5 million Japanese were estimated to have diabetes mellitus in 2012 [2]. Health economics is becoming a major issue; for example, 44.2% of patients on dialysis have diabetic nephropathy [3]. Patient empowerment has recently been proposed as an important concept in self-management for effective glycemic control, whereby diabetes patients set their own glycemic control targets, assume responsibility for their behaviors, and face up to their problems [4]. In Japan, healthcare professionals are attempting to educate patients on maintaining appropriate control of blood sugar levels by promoting behavioral changes based on the concept of better patient empowerment. Empowerment is both a process in which an educational intervention increases the learner’s ability to think critically and act autonomously and an outcome in which an enhanced sense of self-efficacy occurs as a result of the process [5]. However, few tools allow frequent and simple evaluation of patient empowerment. Therefore, we sought to develop a valid and reliable questionnaire for rapidly assessing empowerment.

We created a Japanese-language version of the Appraisal of Diabetes Scale (ADS) [6], a questionnaire that was developed to evaluate a patient’s awareness of the psychological burden of diabetes mellitus and their ability to manage this burden. We then investigated the reliability and validity of this Japanese-language questionnaire, which used three scales. The results showed good correlations between the scale for psychological impact of diabetes mellitus and the effectiveness of dietary therapy, exercise therapy, and pharmacotherapy with hypoglycemic agents or insulin [7]. Subsequently, we developed a Japanese-language version of the Diabetes Family Behavior Checklist (DFBC), which measures family support for patients with diabetes mellitus. When this reliable and valid checklist was used for patients with type 2 diabetes mellitus, glycemic control was found to be strongly influenced by negative family support and a family’s critical opinions of the patient [8].

In the current study, a simple self-completed questionnaire was created for type 2 diabetes mellitus patients living with family to comprehensively evaluate their empowerment, based on self-managed dietary/exercise behaviors, psychological impact, and family support. The reliability and validity of this new questionnaire were tested, and factors relating to patient empowerment were analyzed.

## Methods

### Patients

The patients enrolled in the study had been diagnosed with type 2 diabetes mellitus at least 6 months previously. They were currently living with family and managing their diabetes mellitus through a combination of oral hypoglycemic agents or insulin and dietary or exercise therapy. The study excluded patients with cognitive impairment, drug-induced diabetes, or secondary diabetes. The questionnaire was implemented between 2007 and 2011 at a total of six sites that were university hospitals, core hospitals, health-check centers, or clinics in Fukuoka Prefecture and Kumamoto Prefecture.

### Producing the patient empowerment questionnaire

The patient empowerment questionnaire comprised 13 questions on background data, such as age, gender, symptoms, and HbA1c; seven questions on self-managed dietary behaviors; and three questions on self-managed exercise behaviors (higher scores indicating better management, as well as the ADS (lower scores indicating better management) and DFBC questionnaires. Our Japanese-language version of the ADS questionnaire used three scales: four questions on the subjective impact of diabetes mellitus, two questions on the sense of self-control regarding diabetes mellitus, and one question on self-efficacy in diabetic control [7]. The DFBC produced by Schafer et al. [9] comprised nine positive feedback questions and seven negative feedback questions. In contrast, seven positive feedback questions and six negative feedback questions were extracted for the Japanese version, after we tested the validity and reliability of a Japanese-language DFBC developed by our group with Japanese patients who had type 2 diabetes mellitus [8]. However, the DFBC includes four questions that patients may be unable to answer, depending on the treatment methods used. Therefore, the patient empowerment questionnaire in this study comprised three negative feedback questions (lower scores indicating better performance) and six positive feedback questions (higher scores indicating better performance) from the Japanese-language DFBC.

### Analytical methods and ethical concerns

Completed questionnaires were given ID numbers after they were collected. Analysis methods, including the *t*-test, chi-squared test, and Mann-Whitney *U* test, were used to explore differences between demographic variables of diabetic patients. In addition, Pearson’s correlation, principal component analysis (PCA), one-way analysis of variance (ANOVA), and interclass correlation analysis were used for internal consistency and functional construct validity, factorial construct validity, concurrent validity, and test-retest reliability, respectively. Finally, stepwise multiple linear regression analysis and one-way ANOVA were used to analyze factors that affect patient empowerment. Stepwise multiple regression analyses were performed with the scores of Self-managed dietary behaviors, Psychological impact of diabetes, Patient-family communication (Negative feedback) and Patient-family communication (Positive feedback) as dependent variables, and Age, Gender (0, male; 1, female), Disease duration, Number of symptoms, HbA1c level, and Therapy as independent variables. The independent variable “Therapy” was converted to continuous variables as follows; 1, diet or exercise therapy; 2, both diet and exercise therapy; 3, oral hypoglycemic agent therapy regardless of diet or exercise therapy; 4, Insulin therapy regardless of diet or exercise therapy; 5, both oral and insulin therapy regardless of diet or exercise therapy. The statistical analyses were performed with JMP ver. 10 and SPSS ver. 21.0, and P values less 0.05 were considered significant. This research was conducted with the approval of the Clinical Ethics Committee of Kurume University School of Medicine. Before performing the research, the objectives and methods of the study were explained to the patients and their family members and written informed consent to participate in the study was obtained.

## Results

### Participants

Of the 384 patients from whom data were collected, 46 were excluded because of incomplete answers on the DFBC questionnaire; thus, a total of 338 patients (88.0% response rate) were included in the data for analysis. Table 1 shows the demographic variables for the patients analyzed. There were 162 males and 176 females, with no significant differences observed between two genders in terms of age or disease duration. In terms of family composition, 78.7% of the patients lived with their spouse (approximately 90% of the males and 70% of the females). Approximately 50% of the patients were treated with insulin alone and around 10% with a combination of insulin and oral medication. The mean and standard deviation of the hemoglobin A1c (HbA1c) level was 7.29 ± 1.08% for males and 7.65 ± 1.66% for females.

**Table 1:** Summary of clinical and demographic variables of diabetes patients (n = 338)

	**Male (n = 162)**	**Female (n = 176)**	**P-value**
Age (years)	64.09 ± 10.76	63.02 ± 11.20	n.s.
≤ 50	19	21	
50 – 59	37	37	
60 – 69	57	69	
70 – 79	38	40	
≥ 80	11	9	
Disease duration (years)	12.92 ± 9.59	12.71 ± 10.01	n.s.
BMI	24.01 ± 4.89	24.64 ± 4.79	n.s.
Living together with (includes multiple answers)			
Spouse	266 (78.7%)		
Sibling(s)	8 (2.4%)		
Children	50 (14.9%)		
Father/Mother	10 (3.0%)		
Other	4 (1.1%)		
Treatment method (includes multiple answers)			
Diet	184 (54.4%)		
Exercise	102 (30.2%)		
Oral Hypoglycemic Agents	208 (61.5%)		
Insulin	156 (46.2%)		
Oral + Insulin	39 (11.5%)		
HbA1c	7.29 ± 1.08	7.56 ± 1.19	0.0158^#^

Continuous data are expressed as mean ± SD.

Statistical analysis was performed by unpaired *t*-test.

Nonparametric data (HbA1c) were analyzed by Mann-Whitney *U*-test.

^#^: A small sized effect was found d = 0.24.

### Internal consistency and construct validity tests

We investigated the internal consistency of the 26 questions extracted from seven scales for the short version of the patient empowerment questionnaire to confirm whether or not the questions were meaningful. Pearson’s correlation coefficients were calculated to assess the relationships between the combined scores for question groups categorized according to the seven scales and the individual scores for each question, and compared with the empirical correlation criterion of 0.4 from Aaronson et al. [10]. The results are shown in Table 2. The coefficient was <0.4 for questions A-1, A-2, and A-6 of the self-managed dietary behaviors scale; questions B-2 and B-6 of the sense of self-control over diabetes scale; and questions D-13 and D-15 of the positive feedback group in the patient-family communication scale. The coefficients for all other questions were >0.4. This suggests that the questions were meaningful with the exception of the seven questions given above. Cronbach’s alpha for each scale ranged between 0.693 and 0.891 for six of the scales, but was 0.552 for the sense of self-control over diabetes scale. Table 2 also shows results after the seven items were deleted. Pearson’s correlation coefficient for each item ranged between 0.412 and 0.846 except for question A-7. Cronbach’s alpha for the five scales ranged between 0.695 and 0.891. This demonstrates that construct validity was sufficiently satisfied for these six scales. The scale for self-efficacy in diabetic control of the ADS questionnaire (question B-5) was excluded because Cronbach’s alpha cannot be calculated from a single question. Ultimately, 18 questions in five scales were selected from among the 31 questions constituting seven scales in the original empowerment questionnaire.

**Table 2:** Construct validity test for the short version of the empowerment questionnaire

	**Pearson’s correlation coefficients**	**Cronbach’s α**	**Pearson’s correlation coefficients**	**Cronbach’s α**
**Self-managed dietary behaviors questionnaire (four-step answers, higher scores are better)**				
Self-managed dietary behaviors scale		0.747		0.778
A-1 Do you eat three meals a day?	0.245		-	
A-2 Are you taking your meals at fixed times every day?	0.325		-	
A-3 Do you follow the meal plans specified by the physician or dietitian?	0.561		0.643	
A-4 Do you observe balanced food intake in your three meals?	0.666		0.701	
A-5 Do you stick to the amounts specified for staple foods?	0.570		0.619	
A-6 Do you eat protein at meals?	0.365		-	
A-7 Do you eat vegetables at all three meals?	0.489		0.390	
**Self-managed exercise behaviors questionnaire (four-step answers, higher scores are better)**				
Self-managed exercise behaviors scale		0.891		0.891
A-8 Do you exercise more than 20 minutes a day?	0.829		0.829	
A-9 Do you remember to exercise even if busy at work or home?	0.846		0.846	
A-10 Do you come up with different ways to exercise when the weather is bad?	0.691		0.691	
**ADS questionnaire (five-step answers)**				
Psychological impact of diabetes scale (lower scores are better)	0.758		0.758	
B-1 How upsetting is having diabetes for you?	0.555		0.555	
B-3 How much uncertainty do you currently experience in your life as a result of being diabetic?	0.617		0.617	
B-4 How likely is your diabetes to worsen over the next several years? (Try to give an estimate based on your personal feeling rather than based on a rational judgment.)	0.502		0.502	
B-7 To what degree does your diabetes get in the way of your developing life goals?	0.553		0.553	
Sense of self-control over diabetes scale (higher scores are better)	0.552			
B-2 How much control over your diabetes do you have?	0.382		-	
B-6 How effective are you in coping with your diabetes?	0.382		-	
Self-effort in diabetic control scale (higher scores are better)				
B-5 Do you believe that achieving good diabetic control is due to your efforts as compared to factors which are beyond your control?	1.000		-	
**DFBC questionnaire (five-step answers)**				
Patient-family communication scale (negative feedback, lower scores are better)	0.735		0.735	
D-4 Criticize you for not exercising regularly?	0.529		0.529	
D-6 Nag you about not following your diet?	0.629		0.629	
D-7 Argue with you about your diabetes self-care activities?	0.553		0.553	
Patient-family communication scale (positive feedback, higher scores are better)	0.693		0.695	
D-1 Praise you for following your diet?	0.454		0.499	
D-3 Suggest things that might help you take insulin on time?	0.427		0.412	
D-9 Plan family activities so that they will fit in with your diabetes self-care schedule?	0.469		0.425	
D-10 Congratulate you for sticking to your diabetes self-care schedule?	0.568		0.588	
D-13 Exercise with you?	0.255		-	
D-15 Buy you things containing sugar to carry with you in case of hypoglycemic reaction?	0.380		-	

### Test-retest reliability

The reproducibility test was performed with the same study subjects, who completed the same questionnaire after a 1-week interval. Although the results are not shown in tabulated form, the correlation coefficients between each of the 18 questions and the total scores for each of the five scales before and after the 1-week interval ranged from 0.575 to 0.762. The significantly high correlations confirmed that the developed questionnaire was reproducible.

### Factorial construct validity

To test the independence of the five scales, PCA was performed to measure the factor loadings for the 18 questions. Table 3A shows results with the highest factor loading shown in bold and italic text for each question. The questions were grouped according to the following principal components: 1) diet, dietary balance, amount eaten, vegetable intake (questions A-3, 4, 5, and 7); 2) exercise amount, exercise habits, exercise type (questions A-8, 9, and 10); 3) the four questions on psychological impact of diabetes (questions B-1, 3, 4, and 7); 4) “Praise you for following your diet?” (question D-1), “Suggest things that might help you take insulin on time?” (question D-3), “Plan family activities so that they will fit in with your diabetes self-care schedule?” (question D-9), “Congratulate you for sticking to your diabetes self-care schedule?” (question D-10); and 5) “Criticize you for not exercising regularly?” (question D-4), “Nag you about not following your diet?” (question D-6), “Argue with you about your diabetes self-care activities?” (question D-7). The grouping for each question was clear, and the groupings were judged to independently measure different dimensions.

**Table 3:** Principal component analysis: does each scale measure a different dimension?

**A. Factorial construct validity**	**n = 338**				
**Factor loading after varimax rotation**	**Principal component**	**Principal component**	**Principal component**	**Principal component**	**Principal component**
	**1**	**2**	**3**	**4**	**5**
A-3 Do you follow the meal plans specified by the physician or dietitian?	*0.822*	0.098	0.084	0.135	-0.007
A-4 Do you observe balanced food intake in your three meals?	*0.828*	0.200	-0.000	0.105	-0.002
A-5 Do you stick to the amounts specified for staple foods?	*0.811*	0.049	-0.011	0.010	-0.130
A-7 Do you eat vegetables at all three meals?	*0.508*	0.261	-0.164	0.022	-0.037
A-8 Do you exercise more than 20 minutes a day?	0.164	0.912	-0.050	0.072	-0.039
A-9 Do you remember to exercise even if busy at work or home?	0.189	0.903	0.004	0.133	-0.057
A-10 Do you come up with different ways to exercise when the weather is bad?	0.174	0.799	-0.034	0.165	-0.096
B-1 How upsetting is having diabetes for you?	-0.128	0.053	0.740	0.031	0.152
B-3 How much uncertainty do you currently experience in your life as a result of being diabetic?	0.068	0.058	0.801	0.007	0.116
B-4 How likely is your diabetes to worsen over the next several years? (Try to give an estimate based on your personal feeling rather than based on a rational judgment.)	-0.177	-0.150	0.715	-0.052	-0.071
B-7 To what degree does your diabetes get in the way of your developing life goal?	0.160	-0.065	0.771	0.054	-0.022
D-4 Criticize you for not exercising regularly?	0.025	-0.099	-0.014	0.135	0.788
D-6 Nag you about not following your diet?	-0.064	-0.077	0.104	0.205	0.799
D-7 Argue with you about your diabetes self-care activities?	-0.124	-0.014	0.093	0.141	0.749
D-1 Praise you for following your diet?	0.058	0.033	0.025	0.781	0.047
D-3 Suggest things that might help you take insulin on time?	-0.013	0.064	-0.074	0.569	0.310
D-9 Plan family activities so that they will fit in with your diabetes self-care schedule?	0.191	0.164	0.067	0.650	0.035
D-10 Congratulate you for sticking to your diabetes self-care schedule?	0.023	0.111	0.012	0.761	0.247
**B. Correlation within the five scales?**	**n = 338**				
**Pearson’s correlation coefficients**	**Self-managed dietary behaviors**	**Self-managed exercise behaviors**	**Psychological impact of diabetes**	**Patient-family communication:**	
				**Positive feedback**	**Negative feedback**
A-3 Do you follow the meal plans specified by the physician or dietitian?	0.643	0.290	0.042	0.197	-0.037
A-4 Do you observe balanced food intake in your three meals?	0.701	0.356	-0.024	0.194	-0.066
A-5 Do you stick to the amounts specified for staple foods?	0.619	0.237	-0.042	0.066	-0.158
A-7 Do you eat vegetables at all three meals?	0.390	0.297	-0.133	0.109	-0.113
A-8 Do you exercise more than 20 minutes a day?	0.329	0.829	-0.085	0.194	-0.109
A-9 Do you remember to exercise even if busy at work or home?	0.361	0.846	-0.038	0.245	-0.108
A-10 Do you come up with different ways to exercise when the weather is bad?	0.330	0.691	-0.070	0.238	-0.118
B-1 How upsetting is having diabetes for you?	-0.104	-0.041	0.555	0.079	0.166
B-3 How much uncertainty do you currently experience in your life as a result of being diabetic?	0.049	0.009	0.617	0.068	0.132
B-4 How likely is your diabetes to worsen over the next several years? (Try to give an estimate based on your personal feeling rather than based on a rational judgment.)	-0.178	-0.169	0.502	-0.092	0.040
B-7 To what degree does your diabetes get in the way of your developing life goal?	0.102	-0.021	0.553	0.048	0.065
D-4 Criticize you for not exercising regularly?	-0.049	-0.110	0.053	0.300	0.529
D-6 Nag you about not following your diet?	-0.106	-0.110	0.148	0.344	0.629
D-7 Argue with you about your diabetes self-care activities?	-0.142	-0.075	0.122	0.280	0.553
D-1Praise you for following your diet?	0.138	0.159	0.033	0.499	0.233
D-3 Suggest things that might help you take insulin on time?	0.047	0.113	-0.014	0.412	0.330
D-9 Plan family activities so that they will fit in with your diabetes self-care schedule?	0.241	0.265	0.049	0.425	0.175
D-10 Congratulate you for sticking to your diabetes self-care schedule?	0.109	0.188	0.036	0.588	0.358

To examine whether or not each question belongs to only one scale, a correlation analysis was performed for all questions. The results are shown in Table 3B. The correlation coefficient for each scale was compared with each question. Diet, dietary balance, amount eaten, and vegetable intake (questions A-3, 4, 5, and 7) show a high level of belonging only to the self-managed dietary behaviors scale; exercise amount, exercise habits, and exercise type (questions A-8, 9, and 10) to the self-managed exercise behaviors scale; “How upsetting is having diabetes for you?” (question B-1)”, How much uncertainty do you currently experience in your life as a result of being diabetic?” (question B-3), “How likely is your diabetes to worsen over the next several years?” (question B-4), and “To what degree does your diabetes get in the way of your developing life goal?” (question B-7) to the psychological impact of diabetes scale; “Criticize you for not exercising regularly” (question D-4), “Nag you about not following your diet” (question D-6), and “Argue with you about your diabetes self-care activities” (question D-7) to the patient-family communication (negative feedback scale); and “Praise you for following your diet” (question D-1), “Suggest things that might help you take insulin on time?” (question D-3), “Plan family activities so that they will fit in with your diabetes self-care schedule?” (question D-9), and “Congratulate you for sticking to your diabetes self-care schedule?” (question D-10) to the patient-family communication (positive feedback scale). The results verified that individual questions belong to a single scale.

### Concurrent validity

Ten diabetes-related symptoms and the HbA1c level were examined to assess the relationship of each scale to external standards. Although the data are not shown, significant differences were observed in whether or not the following symptoms occurred: “cold sweats” due to transient hypoglycemia in the self-managed dietary behaviors scale; “easily fatigued”, “easily out of breath”, and “irritable” in the self-managed exercise behaviors scale; and “swelling”, “bad mood”, “reduced vision”, “easily fatigued”, “unable to sleep”, and “irritable” in the psychological impact of diabetes scale. A significant difference between good or poor glycemic control (HbA1c ≤6.8% or ≥6.9%) was also observed in the psychological impact of diabetes scale. These results demonstrate that the scales for self-managed dietary and exercise behaviors give sensitive measurements of the physiological aspects of diabetes patients, whereas the psychological impact of diabetes scale allows sensitive measurement of the psychological aspects, including mood, sleep, emotional symptoms, and good/poor glycemic control. There was no significant difference in each diabetes mellitus-related symptom in the scales for patient-family communication (positive feedback and negative feedback).

Next, the relationship between scale scores and each diabetes therapy was examined (Table 4). Different treatment groups had significantly better scores as follows: patients using dietary therapy had better scores on the scales for self-managed dietary behaviors, patient-family communication (positive feedback), and HbA1c; patients using exercise therapy had better scores on the scales for self-managed dietary behaviors, self-managed exercise behaviors, psychological impact of diabetes, and patient-family communication (negative feedback); and patients using oral hypoglycemic therapy had better scores on the scale for psychological impact of diabetes. In contrast, for patients who had been undergoing insulin therapy, although a significant difference between the treatment and non-treatment groups was seen in the psychological impact of diabetes scale and HbA1c, the non-treatment group showed better scores than the treatment group. These results suggest that the insulin treatment group includes many patients with poor glycemic control. Therefore, the data were reanalyzed after the stratification of the patients within the insulin treatment group according to good or poor glycemic control (HbA1c ≤6.8% or ≥6.9%). Scores for the psychological impact of diabetes scale were 10.83 ± 3.28 in the good glycemic control subgroup (n = 41) and 12.10 ± 2.63 in the poor glycemic control subgroup (n = 115). Therefore, the psychological impact is significantly lower in patients with good glycemic control (P < 0.0284; data not shown).

**Table 4:** Comparison of scale scores between the non-treatment and the four treatment groups

**Therapy**	**Diet**			**Exercise**			**Hypoglycemic agents**			**Insulin**		
**Scale**	**Treatment**	**Non-treatment**	**P-value**	**Treatment**	**Non-treatment**	**P-value**	**Treatment**	**Non-treatment**	**P-value**	**Treatment**	**Non-treatment**	**P-value**
	**n = 184**	**n = 154**	**(effect size)**	**n = 102**	**n = 236**	**(effect size)**	**n = 208**	**n = 130**	**(effect size)**	**n = 156**	**n = 182**	**(effect size)**
Self-managed dietary behaviors^1)^	13.79 ± 2.05	12.26 ± 2.79	< 0.01 (d = 0.63)	14.03 ± 2.04	12.69 ± 2.61	< 0.01 (d = 0.55)	13.12 ± 2.52	13.05 ± 2.56	0.83 (d = 0.03)	13.14 ± 2.46	13.04 ± 2.59	0.71
Self-managed exercise behaviors^2)^	7.88 ± 3.14	7.28 ± 3.00	0.07 (d = 0.20)	9.35 ± 2.42	6.85 ± 3.04	< 0.01 (d = 0.87)	7.65 ± 3.04	7.53 ± 3.16	0.72 (d = 0.04)	7.56 ± 3.16	7.64 ± 3.03	0.82
Psychological impact of diabetes^3)^	10.89 ± 3.42	11.16 ± 2.72	0.43 (d = 0.09)	10.36 ± 3.42	11.30 ± 2.94	0.01 (d = 0.30)	10.57 ± 3.16	11.72 ± 2.92	< 0.01 (d = 0.37)	11.77 ± 2.86	10.37 ± 3.19	< 0.01 (d = 0.46)
Patient–family communication:												
Positive feedback^4)^	8.98 ± 4.38	8.01 ± 3.71	0.03 (d = 0.24)	9.10 ± 4.55	8.30 ± 3.90	0.10 (d = 0.19)	8.63 ± 4.25	8.40 ± 3.90	0.62 (d = 0.06)	8.72 ± 4.03	8.39 ± 4.19	0.47 (d = 0.08)
Patient–family communication:												
Negative feedback^5)^	5.28 ± 2.78	5.37 ± 2.90	0.78 (d = 0.03)	4.81 ± 2.49	5.54 ± 2.95	0.03 (d = 0.26)	5.21 ± 2.86	5.51 ± 2.78	0.34 (n = 0.11)	5.34 ± 2.74	5.31 ± 2.92	0.92
HbA1c (%)	7.29 ± 1.00	7.59 ± 1.28	0.02 (d = 0.26)	7.41 ± 0.98	7.44 ± 1.21	0.78 (d = 0.03)	7.44 ± 1.09	7.42 ± 1.24	0.52 (d = 0.02)	7.57 ± 1.16	7.31 ± 1.13	0.04

1), 2), 4): Higher scores are better; 3), 5): Lower scores are better.

Data are expressed as mean ± SD.

### Factors that affect patient empowerment

**Figure 1:**
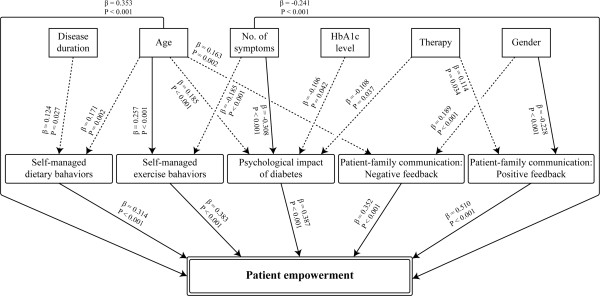
**Multiple linear regression analyses.** Firstly, stepwise multiple linear regression analyses were performed with the scores for Self-managed dietary behaviors, Self-managed dietary behaviors, Psychological impact of diabetes, Patient-family communication (Negative feedback) and Patient-family communication (Positive feedback) as dependent variables; and Age, Gender (0, male; 1, female), Disease duration (years), Number of symptoms (0 - 10), HbA1c level (%, NGSP), and Therapy (1 - 5) as independent variables. Scores for the dependent variables “Psychological impact of diabetes” and “Patient-family communication (Negative feedback) were reversed to “Higher scores are better”. The independent variable “Therapy” was converted to continuous variables as follows; 1, diet or exercise therapy; 2, both diet and exercise therapy; 3, oral hypoglycemic agent therapy regardless of diet and exercise therapy; 4, Insulin therapy regardless of diet and exercise therapy; 5, both oral and insulin therapy regardless of diet and exercise therapy. Secondly, stepwise multiple linear regression analysis was performed with the scores of Patient empowerment and Age, Gender, Disease duration, Number of symptoms, HbA1c level, and Therapy as independent variables. Finally, stepwise multiple linear regression analysis was performed with the scores for Patient empowerment as dependent variables and Self-managed dietary behaviors, Psychological impact of diabetes, Patient-family communication (Negative feedback) and Patient-family communication (Positive feedback) as independent variables. The independent variables with standardized partial regression coefficients >0.2 or < -0.2 are presented as solid arrows.

To clarify the factors affecting patient empowerment as measured by the developed questionnaire, stepwise multiple linear regression analysis was performed by using six demographic variables (gender, age, disease duration, number of diabetes-related symptoms, HbA1c level, and therapy) as independent variables and the five scales as dependent variables (Figure 1). Disease duration and age affected the self-managed dietary behaviors scale; age and number of diabetes-related symptoms affected the self-managed exercise behaviors scale; age, therapy, number of diabetes-related symptoms, and HbA1c level affected the psychological impact of diabetes scale; gender and therapy affected the patient-family communication (positive feedback scale); and gender and age affected the patient-family communication (negative feedback scale). Because age, gender, and number of symptoms had a strong impact on patient empowerment, the patients were stratified according to age or gender. The results showed a significant difference by gender in the scales for psychological impact of diabetes, patient-family communication (positive feedback), and patient-family communication (negative feedback). Age had an effect on all scales apart from patient-family communication (positive feedback); the scores in the four scales that showed an effect were better for older patients (Table 5).

**Table 5:** Stratification of the five scale scores by gender and age

	**Scale**	**Self-managed dietary behaviors**^**1)**^	**Self-managed exercise behaviors**^**2)**^	**Psychological impact of diabetes**^**3)**^	**Patient-family communications:**	
					**Positive feedback**^**4)**^	**Negative feedback**^**5)**^
Gender	Male (n = 162)	12.90 ± 2.70	7.67 ± 2.97	10.51 ± 2.95	9.45 ± 4.27	5.86 ± 2.99
	Female (n = 176)	13.27 ± 2.36	7.55 ± 3.19	11.48 ± 3.20	7.70 ± 3.78	4.83 ± 2.59
				P < 0.0043	P < 0.0001	P = 0.0008
Age	≤ 50 (n = 40)	12.83 ± 2.15	6.10 ± 2.90	11.73 ± 3.06	8.35 ± 4.29	6.10 ± 3.19
	50 – 59 (n = 74)	12.24 ± 2.47	7.31 ± 3.09	11.41 ± 2.95	8.18 ± 3.72	5.65 ± 3.03
	60 – 69 (n = 126)	12.98 ± 2.63	7.58 ± 3.12	11.19 ± 3.14	8.23 ± 3.89	5.42 ± 3.00
	70 – 79 (n = 78)	13.99 ± 2.22	8.47 ± 2.90	10.45 ± 3.12	9.29 ± 4.64	4.65 ± 2.12
	≥ 80 (n = 20)	14.00 ± 2.77	8.50 ± 2.72	9.25 ± 3.01	9.30 ± 4.23	4.55 ± 2.14
	One-way ANOVA	P = 0.0002	P = 0.0010	P = 0.0125	P = 0.3212	P = 0.0397

1), 2), 4): Higher scores are better; 3), 5): Lower scores are better.

Data are expressed as mean ± SD.

## Discussion

### Development of a novel and short version of a patient empowerment questionnaire

Patients with diabetes mellitus must make significant changes in diet, other daily habits, and lifestyles. The circumstances for each patient can also differ significantly in terms of family relationships, social environment, and economic situation. These factors may prevent patients from responding to a one-size-fits-all intervention to increase patient empowerment. Self-management of diet and exercise provide the foundations for glycemic control by patients with diabetes [11]. However, problem-solving skills, the patient’s psychosocial state, and family relationships are also important; the key to maintaining a high quality of life may lie in the rhythm of daily activities and family communication [12-14]. On the basis of our experience to date, we developed a short and self-completed patient empowerment questionnaire that combines the ADS questionnaire, which measures the psychological state of diabetes patients; the DFBC questionnaire, which measures the state of relationships between patients and their families; and questionnaires that measure self-managed behaviors for diet and exercise. The questionnaire was trialed with 338 male and female type 2 diabetes patients living with their families, and the contents were streamlined to a total of 18 questions from the five scales of self-managed dietary behaviors, self-managed exercise behaviors, psychological impact of diabetes, and positive as well as negative feedback in patient-family communication. This questionnaire was tested for reliability and validity, and the results were satisfactory for the measurement of the empowerment of patients with diabetes mellitus. An analysis of how the results from the five scales changed for different therapies revealed significant differences between the treatment and non-treatment groups for two scales (including self-managed dietary behaviors) and the HbA1c of patients receiving dietary therapy, four scales, but not patient–family communication (positive feedback) for patients receiving exercise therapy, and the psychological impact of the diabetes scale for patients receiving oral hypoglycemic therapy. These results, which reflect the particular characteristics of each therapy, suggest that the questionnaire shows better sensitivity. However, patients undergoing insulin therapy included those with low empowerment compared with those not receiving insulin therapy, which led to results different from results with other therapies. This suggests that when comparing treatment methods with this questionnaire, possible differences in the characteristics of the patients included in the different groups should be considered.

Anderson proposed the concept of patient empowerment and developed a scale to measure psychosocial self-efficacy [15]. This questionnaire comprised 28 questions with three scales on managing the psychosocial aspects of diabetes, assessing dissatisfaction and readiness to change, and setting and achieving diabetes goals. The questionnaire satisfied concurrent validity in comparison with the diabetes care profile [16] and educational level and has been reported to be useful when evaluating educational and psychosocial interventions for diabetes mellitus patients. However, the validity and reliability of the diabetes care profile remain to be demonstrated. Anderson’s concept has been accepted in Japan and trialed in various types of patient education programs to improve empowerment; however, questionnaires of proven validity and reliability for evaluating outcomes have not yet been developed. Compared with Anderson et al.’s questionnaire, our short version for Japanese patients with type 2 diabetes mellitus consisted of five scales but fewer questions, including seven about patient-family relationships. For patients with diabetes mellitus who focus on daily diet and exercise habits, family support is a vital factor in maintaining good glycemic control over the long term. Diabetes mellitus differs from other chronic diseases in this respect. Results from a study of Japanese patients with type 2 diabetes mellitus show that family support at mealtimes is an important factor for improving glucose metabolism and self-managed behaviors, particularly for patients aged 60 years and above [17]. Wen et al. showed that family support can be a major barrier to dietary self-management in a study that used the family support DFBC for elderly Hispanic patients with type 2 diabetes mellitus [13]. They also suggested an important function for family support, especially for elderly patients.

### Analysis of factors that affect diabetes patient empowerment

To assess the factors that affect patient empowerment, stepwise multiple linear regression analysis was performed with six demographic variables (gender, age, disease duration, number of diabetes-related symptoms, HbA1c level, and therapy) as independent variables and five scales as dependent variables. Disease duration and HbA1c level each affected one scale; number of symptoms, therapy, and gender each affected two scales; and age affected four scales. Furthermore, the number of symptoms, age, and gender moderately affected the scales for the psychological impact of diabetes, self-managed exercise behaviors, and patient-family communication (positive feedback), respectively. Stratification of patients by gender revealed significant differences between male and female scores in the scales for the psychological impact of diabetes, patient-family communication (negative feedback) and patient-family communication (positive feedback). Scores were better for females for patient-family communication (negative feedback) and for males for the psychological impact of diabetes and patient-family communication (positive feedback). Diabetes-related symptoms did not affect scores on the five scales for males, but significantly worsened scores on the psychological impact of diabetes score of female patients with diabetes-related symptoms compared with those without symptoms. This suggests that gender has a major impact on patient empowerment and that female patients may face more psychological or family communication problems. Possible factors that should not be ignored include postmenopausal decline in physical function and general malaise specific to females. Furthermore, the gender differences were observed in scores regarding patient-family communication. Most family members living with diabetes mellitus patients in this study were spouses. This result may reflect spousal relationships and the typical division of roles between males and females in Japanese culture, whereby females mainly assume responsibility for preparing and serving daily meals or communicating within the family. As discussed above, Watanabe et al. also found that patients under the age of 60 years who received family support had significantly lower HbA1c levels than those who did not. They reported that male patients in particular showed better glycemic control if they received support for meals/light snacks rather than suggestions or encouragement from family members [17]. Chiu et al. also analyzed functional limitations according to gender differences in a cohort of adult patients with type 2 diabetes mellitus, using biobehavioral and psychosocial indicators [18]. Their results showed that female patients had better self-managed behaviors for meals and blood glucose management, but male patients were better in terms of BMI, HbA1c level, blood pressure, early complications, exercise behavior, understanding of the importance of glycemic control, self-efficacy, coping, depressive symptoms, and family support. These results revealed that biological and behavioral factors have a direct impact on functional limitations. Therefore, the results suggest that the interventions to improve the empowerment of females need to consider biological and behavioral functional limitations specific to females.

In this study, patient empowerment was related to aging for both males and females. No detailed research has been conducted on the relationship between age and empowerment of diabetes mellitus patients. This study enrolled patients living with family (in most cases a spouse). We surmised that the more time older patients had at their disposal (the retirement age in Japan is 60–65 years), the easier it was for them to self-manage diet and exercise, and thereby improve family relationships. Therefore, the psychological impact of diabetes appeared to decrease with age and ultimately improve patient empowerment. A study of Japanese patients revealed that glycemic control was more strongly affected by family support in younger patients and that this impact diminished in older patients [17]. More detailed information will be needed to clarify the relationship between aging and empowerment of diabetes mellitus patients.

When measuring and assessing patient empowerment, the ideal questionnaire should be able to measure not only problem-solving and self-management skills, but also functional/psychological aspects and family support, which are affected by gender, age, and disease-related symptoms. We developed a short, self-completed questionnaire that contains 18 questions in five scales on self-managed behaviors for diet/exercise, psychological impact of diabetes, and patient–family communication and that shows better reliability and validity. Additionally, this questionnaire may help prevent complications of diabetes mellitus by improving patient empowerment and in turn reducing social burdens (e.g., health economics) due to diabetes mellitus. Our results suggest that the questionnaire is a useful tool for comprehensively measuring the empowerment of individual patients and evaluating the impact of symptoms and therapies on empowerment.

## Abbreviations

ADS: Appraisal of diabetes scale; DFBC: Diabetes family behavior checklist; HbA1c: Glycated hemoglobin.

## Competing interests

The authors declare that they have no competing interests.

## Authors’ contributions

YH lead in developing this manuscript participated in study design, data collection and drafted manuscript. SI, AO, YT, HN, and TK participated in data collection and discussion. MN was involved in data editing, statistical analysis, interpretation, manuscript preparation, and provided discussion and advice. KT and RB actively provided discussion and advice. YI supervised YH, was participated in study design, data editing, analysis, manuscript preparation, and also supervised all aspects of this study. All authors read and approved the final manuscript.

## References

[B1] IDF Diabetes Atlas Sixth Edition http://www.idf.org/diabetesatlas

[B2] Ministry of Health, Labour and Welfare Overview of the National Health and Nutrition Survey results 2012 http://www.mhlw.go.jp/file/04-Houdouhappyou-10904750-Kenkoukyoku-Gantaisakukenkouzoushinka/0000032813.pdf

[B3] The Japanese society for dialysis therapy, basic statistics on chronic dialysis patients 2012 http://docs.jsdt.or.jp/overview/pdf2012/p11.pdf

[B4] Anderson RM Funnell MM Patient empowerment: reflections on the challenge of fostering the adoption of a new paradigm Patient Educ Couns 2005 57 2 153 157 10.1016/j.pec.2004.05.008 15911187

[B5] Anderson RM Funnell MM Patient empowerment: myths and misconceptions Patient Educ Couns 2010 79 277 282 10.1016/j.pec.2009.07.025 19682830 PMC2879465

[B6] Carey MP Jorgensen RS Weinstock RS Sprafkin RP Reliability and validity of the appraisal of diabetes scale J Behav Med 1991 14 1 43 47 10.1007/BF00844767 2038044

[B7] Hara Y Koyama S Morinaga T Ito H Kohno S Hirai H Kikuchi T Tsuda T Ichino I Takei S Yamada K Tsuboi K Breugelmans R Ishihara Y The reliability and validity of the Japanese version of the appraisal of diabetes scale for type 2 diabetes patients Diab Res Clin Prac 2011 91 1 40 46 10.1016/j.diabres.2010.09.034 21040993

[B8] Hara Y Iwasita S Ishii K Inada C Okada A Tajiri Y Nakayama H Kato T Nishida K Ogata Y Omori H Morinaga T Yamaguchi M Nakao M Tsuboi K Breugelmans R Ishihara Y The reliability and validity of the Japanese version of the diabetes family behavior checklist for assessing the relationship between type 2 diabetes mellitus patients and their families with respect to adherence to treatment Diab Res Clin Prac 2013 99 1 39 47 10.1016/j.diabres.2012.10.014 23107110

[B9] Schafer LC Kevin MS McCaul D Glasgow RE Supportive and non-supportive family behaviors: relationships to adherence and metabolic control in persons with type I diabetes Diab Care 1986 9 2 179 185 10.2337/diacare.9.2.179 3698784

[B10] Aaronson NK Ahmedzai S Bergman B Bullnger M Cull A Duez NJ Filiberti A Flechtner H Fleishman SB De Haes JCJM Kaasa S Klee M Osoba D Razavi D Rofe PB Shraub S Sneeuw K Sullivan M Takeda F The European Organization for Research and Treatment of Cancer QLQ-C30: a Quality-of-life instrument for use in international clinical trials in oncology JNCI 1993 85 5 365 376 10.1093/jnci/85.5.365 8433390

[B11] Dye CJ Haley-Zitlln V Willoughby D Insights from older adults with type 2 diabetes: making dietary and exercise change Diab Educ 2003 29 1 116 127 10.1177/014572170302900116 12632690

[B12] Heisler M Krein SL Smith D Kerr EA Hayward RA How well do patients’ assessments of their diabetes self-management correlate with actual glycemic control and receipt of recommended diabetes services? Diab Care 2003 26 3 738 743 10.2337/diacare.26.3.738 12610031

[B13] Wen LK Parchman ML Shepherd MD Family support and diet barriers among older Hispanic adults with type 2 diabetes Fam Med 2004 36 6 423 430 15181555

[B14] Trento M Bajardi M Passera P Cavallo F Borgo E Porta M Tomalino M A 5-Year randomized controlled study of leaning, problem solving ability, and quality of life modifications in people with type 2 diabetes managed by group care Diabs Care 2004 27 3 670 675 10.2337/diacare.27.3.670 14988283

[B15] Anderson RM Funnell MM Fitzgerald JT Marrero DG The diabetes empowerment scale: a measure of psychosocial self-efficacy Diab Care 2000 23 6 739 743 10.2337/diacare.23.6.739 10840988

[B16] Fitzgerald JT Davis WK Connell CM Hess GE Funnell MM Hiss RG Development and validation of the diabetes care profile EHP 1996 19 2 208 230 10.1177/016327879601900205 10186911

[B17] Watanabe K Kurose T Kitatani N Yabe D Hishizawa M Hyo T Seino Y The role of family nutritional support in Japanese patients with type 2 diabetes mellitus Intern Med 2010 49 11 983 989 10.2169/internalmedicine.49.3230 20519813

[B18] Chiu CJ Wray LA Gender differences in functional limitations in adults living with type 2 diabetes: behavioral and psychosocial mediators Ann Behav Med 2011 41 1 71 82 10.1007/s12160-010-9226-0 20827519

